# Incidence, Outcomes, and Factors Associated with Intra-Abdominal Hypertension and Primary Abdominal Compartment Syndrome in Abdominopelvic Injury Patients

**DOI:** 10.1155/2020/1982078

**Published:** 2020-08-17

**Authors:** Amonpon Kanlerd, Krissada Nakornchai, Karikarn Auksornchart, Warapan Watkwaw

**Affiliations:** ^1^Unit of Trauma and Surgical Critical Care, Division of General Surgery, Department of Surgery, Faculty of Medicine, Thammasat University, Bangkok, Thailand; ^2^Registered Nurse, Surgical Intensive Care Units, Thammasat University Hospital, Khlong Nueng, Thailand

## Abstract

**Background:**

The primary aim was to identify the incidence of intra-abdominal hypertension (IAH) and primary abdominal compartment syndrome (1^o^ACS) of abdominopelvic injury patients at Thammasat University Hospital (TUH), Thailand, and the secondary objective was to evaluate those factors that contributed to developing these conditions.

**Methods:**

The retrospective cohort of 38 abdominopelvic injury cases was admitted to the intensive care unit at Thammasat University Hospital, from January 1^st^ to December 31^st^, 2018. The bladder pressure was recorded every 4 hours until the urethral catheter was removed. Data of age, gender, weight, height, body mass index, injury mechanisms, initial vital signs, imaging, laboratory data, blood component requirements, abdominal organs involved, treatments including surgery and intervention radiology, abbreviated injury scale (AIS) and injury severity score (ISS), length of ICU stays, and results of treatment were all analyzed.

**Results:**

The patients were mostly young (mean age 31.5 years), male (68.4%), and suffering from blunt trauma (89.5%). The mean maximum bladder pressure was 8.3 ± 5.2 mmHg. Six patients (15.8%) developed IAH, and one patient (2.6%) was diagnosed with 1^o^ACS. Two patients expired. The multivariate analysis showed the patient who had initial Cr ≥ 1.5 g/dL, lower extremity including pelvis AIS ≥3, and ISS >15 was significantly associated with the developing of IAH.

**Conclusions:**

The incidence of IAH and 1^o^ACS was 15.8% and 2.6%. Predicted factors to find developing IAH were initial Cr ≥ 1.5 g/dL, lower extremity AIS ≥3, and ISS >15. We should consider awareness of IAH and 1^o^ACS in abdominopelvic injury patients.

## 1. Introduction

Intra-abdominal hypertension (IAH) is defined as a sustained elevated level of intra-abdominal pressure (IAP) more than 12 mmHg. Abdominal compartment syndrome (ACS) is defined as a sustained raised level of IAP more than 20 mmHg with or without abdominal perfusion pressure (APP) less than 60 mmHg and developing of new end-organ failure. Primary ACS (1^o^ACS) is defined as ACS developing from intra-abdominal pathologies. Secondary ACS (2^o^ACS) means ACS following from extra-abdominal pathologies. And recurrent ACS is defined as redeveloped ACS following medical or surgical treatments of 1^o^ or 2^o^ACS [1].

ACS is caused by persistent increase in IAP leading to a decrease in venous return and cardiac output, and then, end-organ damage occurs [2]. Many reports define both IAH and ACS as significantly associated with morbidity and mortality in critically ill patients [3–5]. The incidence of IAH and ACS varies by the population group; the overall incidence is 30–70% and 10–35%, respectively [6]. If focusing on a trauma patient group, the incidence of IAH is 2–50%, and the incidence of ACS is 0.6–36% [7].

The indirect intra-abdominal pressure measurement uses a catheter inserted into the intraluminal cavity of an intra-abdominal organ and measures the intraluminal pressure [8]. Nowadays, intravesicular pressure measurement has become the gold standard in the analysis of IAP; it has shown strong correlations to the direct IAP measurement and is reliable [2, 9]. The standard recommendations to evaluate IAP mostly use the World Society of the Abdominal Compartment Syndrome (WSACS) recommendation 2013. They recommend measuring IAP at least every 4 hours in an injured patient with any known risk factors for IAH/ACS [1].

This study focuses on IAH and 1^o^ACS from abdominopelvic injury etiology. We want to know precisely the incidence of these conditions in our institute and which factors contribute to development of IAH/1^o^ACS and also what is the clinical result of these conditions. These data may represent the incidence in specific population and guide us to improve the accuracy of protocol.

## 2. Materials and Methods

This retrospective cohort study was conducted at Thammasat University Hospital (TUH), Pathumthani, a tertiary care center located in the central region of Thailand. The study protocol was approved by the Human Ethics Committee of Thammasat University No. 1 (Faculty of Medicine), Thailand, which also waived the requirement for informed consent due to the retrospective nature of the study.

All adult abdominopelvic injury patients (age ≥ 18 years old) admitted to the intensive care unit (ICU) from January 1^st^ to December 31^st^, 2018, were included. The exclusion criteria were the patient who could not insert a urethral catheter for any reason and the patient who had a history of pelvic mass bringing compression to the bladder or previous bladder surgery. Data regarding age, gender, weight, height, body mass index (BMI), injury mechanisms, initial vital signs, clinical presentations, imaging, laboratory data, blood component requirements in the first 24 hours, abdominal organs involved, all recorded intravesicular pressure, urethral catheter complications, treatments including surgery and intervention radiology, regional abbreviated injury scale (AIS), injury severity score (ISS), length of ICU stays, and results of treatment (death or alive until discharged) were all collected and analyzed.

Abdominopelvic injury is defined as any injury to internal organ of abdomen and abdominal wall. The boundary of the abdominopelvic region is below the diaphragm to the pelvic outlet. The injury includes intraperitoneal organs, extraperitoneal organs, pelvic bone, or pelvic viscera. The patient may or may not have had any combined injuries.

All of the patients were initially evaluated at the emergency room (ER) and fitted with a urethral catheter to evaluate the initial intravesicular pressure. The patient who had indication for immediate surgery needed to proceed to the operating room (OR). If the patient did not have indication for surgery, the patient was admitted to ICU for nonoperative management and to intermittent measurement and recording of intravesicular pressure in mmHg every 4 hours. The measurement of IAP is defined by first emptying the bladder and then filling by a maximum of 25 ml of sterile saline solution via the catheter. The zero is referenced at the midaxillary line, and then, the catheter is held vertically above the patient. The end-expiration fluid level was measured in cmH_2_O and converted to mmHg by using this formula: 1 mmHg = 1 cmH_2_O *x* 0.735. The patient who had intravesicular pressure more than 12 mmHg was treated by turning the patient into a supine position and then giving adequate pain control medication. We optimized the organ perfusion and avoided excessive fluid resuscitation. If intravesicular pressure was more than 20 mmHg, we defined the patient as IAH grade III and considered evaluating end-organ failure for diagnosis of 1^o^ACS. The patients diagnosed with 1^o^ACS were treated with decompressive laparotomy.

The primary end point of this study was the patient had abdominopelvic operation which was the end of 1^o^ACS definition, or the urethral catheter was removed. The secondary end point was the length of ICU stays, total blood components required, total operations required, the need for intervention radiology, and mortality.

The statistical analysis tools used were IBM^®^ and SPSS^®^, Version 20 for Mac^®^ (SPSS, Chicago, Illinois, USA). Values were reported as percentages for categorical variables and mean (range) or mean with standard deviation (SD) for continuous variables. Comparison data, such as demographic and clinical characteristics, were evaluated using bivariable analysis. Correlation analysis of qualitative variables to developing IAH/1^o^ACS was using the Pearson Chi-square test and unpaired *t*-test for quantitative variables. Independent risk factors associated with developing IAH/1^o^ACS were measured using a stepwise logistic regression method. Statistical significance was set at *p* < 0.050.

## 3. Results

A total of 38 consecutive abdominopelvic injury cases were identified in the one year. The mean age was 31.5 years (18–67). Most of the patients were male (68.4%). Thirty-four patients (89.5%) were suffering from a blunt mechanism. The mean patient weight, height, and BMI were 61.2 ± 11.3 kg, 1.67 ± 0.09 m, and 21.7 ± 2.8 kg/m^2^, respectively. On primary surveys, seven patients (18.4%) had airway problems. Six cases (15.8%) had ventilation problems, and ten patients (26.3%) were suffering from hemorrhagic shock. Three patients (7.9%) had a Glasgow Coma Scale (GCS) less than 13, and only one patient (2.6%) developed hypothermia. Secondary surveys showed 17 cases (44.7%) had abdominal distension. Three patients (7.9%) developed generalized peritonitis. Nine cases (23.7%) had abdominal wounds, and 2 of them (5.3%) had internal organ evisceration. Initial mean arterial pressure (MAP) was 88 ± 16 mmHg. Initial pulse rate (PR) was 101 ± 18 bpm. Twenty-eight cases (73.7%) had normal chest film. Sixteen patients (42.1%) were positive focused assessment sonography in trauma (FAST). Twenty-eight cases (73.7%) proceeded to computed tomography (CT) of the abdomen, and 26 cases (68.4%) had positive results. Six patients (15.8%) presented with gross hematuria. Most of the patients (52.6%) had single abdominopelvic organ injury, and three patients (7.9%) had four-organ injuries. Four patients (10.5%) had a pelvic fracture. Three patients (7.9%) had urinary bladder injury, but no one had a ruptured bladder. The mean initial IAP was 7.6 ± 4.5 mmHg. The initial hemoglobin (Hb) level was 12.8 g/dL (8.3–16.3), and the platelets count was 248,579 cells/cu.mm (146,000–378,000). The initial creatinine level (Cr) was 1.09 ± 0.34 g/dL, and serum bicarbonate level (HCO_3_) was 21.02 ± 3.79 mmol/L. The international normalized ratio (INR) was 1.11 ± 0.15. Most of the cases (52.6%) had minor head AIS, and 65.8% had minor chest AIS. For abdominal including pelvic AIS, 6 cases (15.8%) had minor injury, 11 cases (28.9%) had moderate injury, 10 cases had serious injury, 10 cases had severe injury, and only 1 case (2.6%) was a critical injury. For lower extremity included pelvic girdle AIS, 24 cases (63.2%) had minor injury, 8 cases (21.1%) had moderate injury, 4 cases (10.5%) had serious injury, and 2 cases (5.3%) had severe injury. The mean ISS was 19 ± 11. The demographic data and patient characteristics are listed in Table 1.

Six cases (15.8%) developed IAP ≥ 12 mmHg and were defined as the IAH group. Only one patient (2.6%) from the IAH grade III group developed renal failure after admission and was defined as 1^o^ACS. Seventeen cases (43.7%) required the operative treatment; 11 cases (28.9%) needed abdominal operations, and 5 patients (13.2%) required orthopedic surgeries (included pelvic bone fixation). Six patients (15.8%) required the angiography with embolization. There were no complications of urethral catheterized and IAP measurement in this study. The mean ICU stay was 4.5 ± 3.3 days. The mean of blood components required in the first 24 hours was 1.2 ± 1.1 unit of packed red cells (PRC), 0.7 ± 1.4 unit of fresh frozen plasma (FFP), and 0.5 ± 1.5 unit of platelets concentration. Two patients (5.3%) died: one from a severe head injury, and the other was multiorgan failure syndrome. The result of treatments is listed in Table 2.

The incidence of IAH and 1^o^ACS from this study was 15.8% and 2.6%, respectively. One patient from the IAH grade III group was diagnosed with blunt abdominal injury and lateral compression-type pelvic fracture. The patient had hemodynamic stability on admission (WSES grade II) and received pelvic angiography with embolization as a primary treatment. The patient subsequently developed oliguria concomitant with gradually increased IAP and serum creatinine in the first day of ICU admission. The maximum IAP was 23 mmHg. The patient was diagnosed with 1^o^ACS and proceeded to decompressive celiotomy. The operative finding was 200 ml of hemoperitoneum, a serosal tear of the transverse colon, and a large zone III retroperitoneal hematoma with a fractured pelvis. Preperitoneal packing with primary repair of the colon was conducted, and then, temporary abdominal closure with negative pressure wound therapy and external pelvic fixation was performed. Unfortunately, the patient expired after this operation with multiorgan failure syndrome. IAH and 1^o^ACS cases are listed in Table 3.

Many variables had a significant difference between the patient who developed IAH and not as shown in Table 1. Some of the variables were selected for a statistical calculation for which factors are associated with developing IAH. The multivariate analysis with stepwise regression analysis was performed, and we found the patient who had initial Cr ≥ 1.5 g/dL, lower extremity including pelvis AIS ≥3, and ISS >15 were statistically significantly associated with developing IAH at *p* < 0.050. The relative risk (RR), 95% confidence interval (95% CI), and the result of multivariate analysis are listed in Table 4. We failed to demonstrate which factors are associated with developing 1^o^ACS because only 1 case developed this condition.

## 4. Discussion

The incidence of IAH in our study was 15.8% (6 cases/year). In past studies [7, 10], the incidence of IAH in mixed trauma populations was 2–50%. Balogh et al. [11] reported the incidence of IAP > 12 mmHg shock/trauma cases who were admitted to ICU was 75%. A study focusing on pelvic fracture patients [12] reported the incidence of IAH was 9.7%. The data of the IAH incidence in our study focus only on abdominopelvic injury patients who were admitted to ICU. This finding of this is more concise coming from a unique population.

Some studies have reported the prevalence and incidence of ACS in trauma patients. One study [13] said the rate of ACS in severe trauma patients was 0–28%, and subgroup analysis in trauma patients admitted to ICU and patients who had visceral injury was 0–5.3% and 1–20%, respectively. Ivatury et al. [14] reported 32% incidence of ACS in severe abdominal trauma, and Balogh et al. [15] reported the incidence of 1^o^ACS in major torso trauma who required shock resuscitation was 6%. Compared to our result, the incidence of 1^o^ACS is 2.6%. The result is different from previous data because of the difference in ACS definitions. In our study, we defined 1^o^ACS as the sustained increase IAP >20 mmHg with new organ dysfunction/failure developing from abdominopelvic injury, but Ivatury et al. [14] defined ACS as a clinical syndrome characterized by increased intra-abdominal pressure (IAP > 25 cmH_2_O) and improvement after abdominal decompression, and they included all type of ACS. Balogh et al. [15] defined 1^o^ACS as the patient having intraperitoneal injuries with IAP > 25 mmHg and progressive organ dysfunction despite resuscitation, which improved after decompression. The true incidence of 1^o^ACS in abdominopelvic injury is still not established although this study may be the first report in this specific population and using the exact 1^o^ACS definitions according WSACS recommendations 2013 [1].

The multivariate analysis shows initial Cr ≥ 1.5 g/dL, lower extremity including pelvis AIS ≥ 3, and ISS > 15 can predict the development of IAH in this study. Many investigators have identified factors contributing to the development of IAH. In the latest prospective multicenter study in mixed critically ill patients with IAH [16], they found BMI ≥ 27 kg/m^2^, APACHE II ≥ 18 points, abdominal distension, and absence of bowel sounds, and PEEP ≥ 7 cmH_2_O was associated with IAH. Mahmood et al. [17] found increased base deficit level, and frequent blood transfusion could predict the developing IAP > 20 mmHg. He L et al. [12] reported only fluid resuscitation over 24 hours was a significant association with IAP > 12 mmHg, and each 1 L increment fluid over 24 hours was significantly associated with an increased 5 mmHg of IAP in fractured pelvis patients. Vatankhah et al. [18] reported risk factors contributed to developing of IAH and ACS in blunt abdominal trauma patients (mixed all age groups). They found the amount of fluid received and pelvis fracture was associated with IAH and ACS but did not evaluate the cut-off point of these factors. From our perspective, we cannot conclude from previous studies which factors contribute to the development of IAH due to variation and mixed population data. This is the strong point of our study because we focus on a unique population, and we make the cut-off points for parameters. These significant parameters correspond with the severity of injuries that means the patient who suffers from severe abdominopelvic injuries has a high risk to develop IAH. The initial Cr ≥ 1.5 g/dL can occur in severe trauma that may develop after exsanguinating, suboptimal fluid resuscitations, renal injuries, or in case of the patient's preexisting conditions. However, a high level of initial Cr in this series mostly associates with severely injured cases. This means that our findings may answer the question of which factors contributed to the development of IAH in adult abdominopelvic injury patients.

About the clinical result of IAH, we found statistically significant differences between both groups in the requirement of blood components in first 24 hours, the need for overall procedures, the need for abdominopelvic operations, orthopedic surgeries, the need for intervention radiology, and ICU stays. Many literatures such as Malbrain et al.' study demonstrated poor clinical outcomes followed IAH [4] and found that the patients who developed IAP > 12 mmHg decreased 30-day survival time. Reintam et al. [16] who studied mixed critically ill patients concluded that a higher IAH grade was associated with a higher mortality. Murphy et al. [19] studied mixed ICU patients and found the patients who developed IAH recorded increased ventilation days, vasoactive medication days, ICU length of stay, death in ICU, and death in hospital and also reported IAH was an independent risk factor of ICU mortality (odds ratio 3.33, 95% CI 1.46–7.57). We cannot conclude which one is the real clinical result of IAH in abdominopelvic injury patients due to the mixed populations of the previous studies. Our study may answer this question.

## 5. Conclusions

The incidence of IAH and 1^o^ACS in our study was 15.8% and 2.6%, respectively. We found that initial Cr ≥ 1.5 g/dL, lower extremity including pelvis AIS ≥3, and ISS >15 are the predictors of developing IAH in abdominopelvic injury patients. Further investigation about the actual clinical outcomes of IAH and 1^o^ACS and strengthening of a useful protocol are our next step.

## Figures and Tables

**Table 1:** Patient characteristics.

Characteristics		Total (*N* = 38)	Non-IAH (*N* = 32)	IAH (*N* = 6)	*p*
Demographic					
Sex (M : F, *n*)		26 : 12	23 : 3	3 : 3	0.290
Age (mean with SD, years)		31.5 ± 12.7	31.4 ± 12.1	32.0 ± 12.0	0.920
Weight (mean with SD, kg)		61.2 ± 11.3	61.3 ± 12.0	60.5 ± 8.1	0.879
Height (mean with SD, m)		1.67 ± 0.09	1.67 ± 0.09	1.68 ± 0.08	0.909
BMI (mean with SD, kg/m^2^)		21.7 ± 2.8	21.8 ± 3.0	21.5 ± 1.8	0.798

Mechanism of injury					
Blunt : penetrating (*n*)		34 : 4	29 : 3	5 : 1	0.593
MVC (*n* (%))		29 (76.3)	25 (78.1)	4 (66.7)	0.545
Fall from the height (*n* (%))		3 (7.9)	2 (6.3)	1 (16.7)	0.385
Body assault (*n* (%))		3 (7.9)	3 (9.4)	0 (0)	0.435

Primary survey					
Airway compromised (*n* (%))		7 (18.4)	5 (15.6)	2 (33.3)	0.305
Breathing and ventilation problem (*n* (%))		6 (15.6)	5 (15.6)	1 (16.7)	0.949
Hemorrhagic shock (*n* (%))		10 (26.3)	6 (18.8)	4 (66.7)	0.014
GCS < 13 (*n* (%))		3 (7.9)	3 (9.4)	0 (0)	0.435
Hypothermia (*n* (%))		1 (2.6)	0 (0)	1 (16.7)	0.019

Initial vital signs					
SBP (mean with SD, mmHg)		116 ± 19	118 ± 16	104 ± 29	0.104
DBP (mean with SD, mmHg)		74 ± 15	76 ± 13	64 ± 21	0.080
MAP (mean with SD, mmHg)		88 ± 16	90 ± 13	77 ± 24	0.076
PR (mean with SD, bpm)		101 ± 18	99 ± 18	113 ± 15	0.073
RR (mean with SD,/min)		21 ± 4	21 ± 4	23 ± 4	0.231
BT (mean with SD, °C)		36.9 ± 0.8	36.9 ± 0.8	36.6 ± 1.1	0.437

Abdominal signs					
Abdominal tenderness (*n* (%))		19 (50)	15 (46.9)	4 (66.7)	0.374
Peritonitis (*n* (%))		3 (7.9)	2 (6.3)	1 (16.7)	0.385
Abdominal distention (*n* (%))		17 (44.7)	11 (34.3)	6 (100)	0.003
Abdominal wounds (*n* (%))		9 (23.7)	7 (21.9)	2 (33.3)	0.545
Flank and back injury (*n* (%))		3 (7.9)	3 (9.4)	0 (0)	0.435
Organ evisceration (*n* (%))		2 (5.3)	1 (3.1)	1 (16.7)	0.173

Initial imaging					
Pelvis fracture (*n* (%))		4 (10.5)	2 (6.3)	2 (33.3)	0.047
Positive FAST (*n* (%))		16 (42.1)	12 (37.5)	4 (66.7)	0.184
Positive abdominal CT (*n* (%))^*∗*^		26 (68.4)	22 (68.7)	4 (66.7)	0.549

Initial laboratory					
Urine analysis					0.887
Microscopic hematuria (*n* (%))		16 (42.1)	13 (40.6)	3 (50)	
Gross hematuria (*n* (%))		6 (15.8)	5 (15.6)	1 (16.7)	
Hct (mean with SD, gm%)		38.9 ± 6.1	39.4 ± 6.3	36.4 ± 4.6	0.271
Hb (mean with SD, g/dL)		12.9 ± 1.8	13.1 ± 1.8	11.8 ± 1.6	0.115
Platelets count (mean with SD, × 10^3^ cells/cu.mm)		249 ± 61	249 ± 60	247 ± 74	0.946
INR (mean with SD)^†^		1.1 ± 0.2	1.1 ± 0.1	1.3 ± 0.2	0.007
Creatinine (mean with SD, g/dL)		1.1 ± 0.3	1.0 ± 0.3	1.5 ± 0.2	0.001
Bicarbonate (mean with SD, mmol/L)		21.0 ± 3.8	21.8 ± 3.4	17.0 ± 3.1	0.003
Base excess (mean with SD, mEq/L)^‡^		−7.3 ± 8.3	−4.2 ± 7.6	−11.0 ± 8.1	0.146
Lactate (mean with SD, mmol/L)^‡^		5.7 ± 4.9	4.2 ± 3.8	7.6 ± 5.7	0.225

Abdominopelvic organ injury					
Diaphragm (*n* (%))		1 (2.6)	1 (3.1)	0 (0)	0.661
Liver (*n* (%))		19 (50)	14 (43.8)	5 (83.3)	0.075
Spleen (*n* (%))		5 (13.2)	3 (9.4)	2 (33.3)	0.111
Kidney (*n* (%))		13 (34.2)	11 (34.3)	2 (33.3)	0.961
Pancreas (*n* (%))		1 (2.6)	0 (0)	1 (16.7)	0.019
Adrenal (*n* (%))		1 (2.6)	1 (3.1)	0 (0)	0.661
Small bowel (*n* (%))		3 (7.9)	2 (6.3)	1 (16.7)	0.385
Colon (*n* (%))		3 (7.9)	1 (3.1)	2 (33.3)	0.012
Mesentery (*n* (%))		5 (13.2)	2 (6.3)	3 (50)	0.004
Bladder (*n* (%))		3 (7.9)	3 (9.4)	0 (0)	0.435
Internal iliac vessel (*n* (%))		1 (2.6)	1 (3.1)	0 (0)	0.661
Abdominal wall (*n* (%))		8 (21.1)	7 (21.9)	1 (16.7)	0.774
Multiple organ injury (*n* (%))		18 (47.4)	13 (40.6)	5 (83.3)	0.055

Abbreviated injury scale (AIS)					
Head AIS (3 : 4 : 5, *n*)		9 : 0 : 1	8 : 0 : 0	1 : 0 : 1	0.090
Face AIS (3 : 4 : 5, *n*)		0 : 0 : 1	0 : 0 : 0	0 : 0 : 1	0.057
Chest AIS (3 : 4 : 5, *n*)		8 : 2 : 0	7 : 2 : 0	1 : 0 : 0	0.721
Abdominopelvic AIS (3 : 4 : 5, *n*)		10 : 10 : 1	8 : 7 : 0	2 : 3 : 1	0.038
Lower extremity and pelvis AIS (3 : 4 : 5, *n*)		4 : 2 : 0	2 : 0 : 0	2 : 2 : 0	0.001
Injury severity scores (ISS) (mean with SD)		19 ± 11	16 ± 7	35 ± 17	<0.001

^*∗*^Ten patients (26.8%) did not proceed to abdominal CT scan; ^†^six patients (15.8%) did not have initial INR result; ^‡^thirteen cases (39.5%) had initial base excess and lactate data.

**Table 2:** The result of treatments.

Variables	Total (*N* = 38)	Non-IAH (*N* = 32)	IAH (*N* = 6)	*p*
IAP result				
Initial IAP (mean with SD, mmHg)	7.6 ± 4.5	6.2 ± 2.4	15.3 ± 5.7	<0.001
Maximum IAP (mean with SD, mmHg)	8.3 ± 5.2	6.4 ± 2.3	18.5 ± 4.5	<0.001
ICU stay (mean with SD, days)	4.5 ± 3.3	3.5 ± 1.2	9.8 ± 5.7	<0.001

Blood components required in 24 hours				
PRC (mean with SD, units)	1.2 ± 1.1	0.7 ± 1.1	3.5 ± 2.3	<0.001
FFP (mean with SD, units)	0.7 ± 1.4	0.4 ± 1.1	2.3 ± 2.0	0.001
Platelets concentration (mean with SD, units)	0.5 ± 1.5	0.1 ± 0.7	2.0 ± 3.3	0.005

Treatment options				
Nonoperative management (*n* (%))	21 (55.3)	20 (62.5)	1 (16.7)	0.038
Operative management (*n* (%))	17 (43.7)	12 (37.5)	5 (83.3)	0.038
Abdominopelvic operations (*n* (%))	11 (28.9)	6 (18.8)	5 (83.3)	0.001
Neurooperations (*n* (%))	2 (5.3)	2 (6.3)	0 (0)	0.529
Chest operations (*n* (%))	2 (5.3)	2 (6.3)	0 (0)	0.529
Orthopedic operations (*n* (%))	5 (13.2)	2 (6.3)	3 (50)	0.004
Intervention radiology (*n* (%))	6 (15.8)	3 (9.4)	3 (50)	0.038

Results				
Alive until discharge (*n* (%))	36 (94.7)	31 (96.9)	5 (83.3)	0.012
Death (*n* (%))	2 (5.3)	1 (3.1)	1 (16.7)	0.173

**Table 3:** Incidence of IAH and 1^o^ACS.

Group	Definitions (mmHg)	Incidence (cases/year)	%
IAH	IAP ≥ 12	6	15.8
Grade I	IAP 12–15	2	5.3
Grade II	IAP 16–20	2	5.3
Grade III	IAP 21–25	2	5.3
Grade IV	IAP > 25	0	0
1^o^ACS	IAP > 20 with new organ dysfunction/failure	1	2.6

**Table 4:** Relative risk (RR) of significant factors for developing of IAH, univariate analysis, and multivariate analysis result.

Variables	RR (95% CI)	Univariate (*p*)	Multivariate (*p*)
Hemorrhagic shock at ER	5.60 (1.21–26.02)	0.014	0.982
Initial MAP ≤ 70 mmHg	0.17 (0.05–0.58)	0.012	0.728
Initial PR ≥ 100 bpm	0.28 (0.04–2.13)	0.169	0.497
Hypothermia at ER	7.40 (3.28–16.72)	0.019	0.272
Abdominal distention	1.55 (1.09–2.20)	0.003	0.954
Pelvis fracture	0.24 (0.06–0.90)	0.047	0.203
Liver injury	0.20 (0.03–1.56)	0.075	0.923
Pancreatic injury	0.14 (0.06–0.31)	0.019	0.068
Colonic injury	0.17 (0.05–0.58)	0.012	0.862
Mesentery injury	0.15 (0.04–0.55)	0.004	0.372
Multiple organs injuries	0.18 (0.02–1.40)	0.055	0.957
INR > 1.2	0.17 (0.04–0.75)	0.009	0.595
Cr ≥ 1.5 g/dL	0.12 (0.04–0.40)	0.001	<0.001
HCO_3_ ≤ 18 mmol/L	0.23 (0.06–0.89)	0.030	0.164
Abdominopelvic AIS ≥ 3	1.40 (1.07–1.84)	0.016	0.369
Lower extremity AIS ≥ 3	0.09 (0.02–0.40)	<0.001	<0.001
ISS >15	1.30(1.05–1.61)	0.070	0.041

## Data Availability

The data that support the findings of this study are available from Thammasat University Hospital, but restrictions apply to the availability of these data, which were used under license for the current study and so are not publicly available. Data are however available from the authors upon reasonable request and with permission of the Faculty of Medicine, Thammasat University, and the Thammasat University Hospital.

## Abbreviations

IAH: Intra-abdominal hypertension
IAP: Intra-abdominal pressure
APP: Abdominal perfusion pressure
1^o^ACS: Primary abdominal compartment syndrome
2^o^ACS: Secondary abdominal compartment syndrome
WSACS: The World Society of the Abdominal Compartment Syndrome
TUH: Thammasat University Hospital
ICU: Intensive care unit
BMI: Body mass index
PRC: Packed red cells
FFP: Fresh frozen plasma
WSES: The World Society of Emergency Surgery
AIS: Abbreviated injury scale
ISS: Injury severity score
ER: The emergency room
OR: The operating room
SD: Standard deviation
MVC: Motor vehicle collision
GCS: Glasgow Coma Scale
SBP: Systolic blood pressure
DBP: Diastolic blood pressure
MAP: Mean arterial pressure
PR: Pulse rate
BT: Body temperature
FAST: Focused assessment sonography in trauma
CT: Computed tomography
Hb: Hemoglobin
Hct: Hematocrit
Cr: Serum creatinine
HCO_3_: Serum bicarbonate
INR: International normalized ratio
BE: Base excess
RR: Relative risk
APACHE II: Acute physiologic and chronic health evaluation II
PEEP: Positive end-expiratory pressure.
